# Phylogeny and source climate impact seed dormancy and germination of restoration-relevant forb species

**DOI:** 10.1371/journal.pone.0191931

**Published:** 2018-02-05

**Authors:** Alexandra E. Seglias, Evelyn Williams, Arman Bilge, Andrea T. Kramer

**Affiliations:** 1 Program in Plant Biology and Conservation, Northwestern University, Evanston, Illinois, United States of America; 2 Department of Plant Science and Conservation, Chicago Botanic Garden, Glencoe, Illinois, United States of America; 3 Department of Statistics, University of Washington, Seattle, Washington, United States of America; 4 Computational Biology Program, Fred Hutchinson Cancer Research Center, Seattle, Washington, United States of America; Università di Pisa, ITALY

## Abstract

For many species and seed sources used in restoration activities, specific seed germination requirements are often unknown. Because seed dormancy and germination traits can be constrained by phylogenetic history, related species are often assumed to have similar traits. However, significant variation in these traits is also present within species as a result of adaptation to local climatic conditions. A growing number of studies have attempted to disentangle how phylogeny and climate influence seed dormancy and germination traits, but they have focused primarily on species-level effects, ignoring potential population-level variation. We examined the relationships between phylogeny, climate, and seed dormancy and germination traits for 24 populations of eight native, restoration-relevant forb species found in a wide range of climatic conditions in the Southwest United States. The seeds were exposed to eight temperature and stratification length regimes designed to mimic regional climatic conditions. Phylogenetic relatedness, overall climatic conditions, and temperature conditions at the site were all significantly correlated with final germination response, with significant among-population variation in germination response across incubation treatments for seven of our eight study species. Notably, germination during stratification was significantly predicted by precipitation seasonality and differed significantly among populations for seven species. While previous studies have not examined germination during stratification as a potential trait influencing overall germination response, our results suggest that this trait should be included in germination studies as well as seed sourcing decisions. Results of this study deepen our understanding of the relationships between source climate, species identity, and germination, leading to improved seed sourcing decisions for restorations.

## Introduction

The next chapter of our environmental history will be defined by ecological restoration (hereafter referred to as ‘restoration’). Urban expansion, development, and the influx of invasive species have led to the need to restore degraded habitats across the globe [1]. Restoration often involves introducing seeds of desired, native species to a disturbed or degraded site, with the goal of establishing a diverse and resilient plant community [2,3]. To accomplish this, restoration practitioners must determine the most appropriate species and seed sources to use at restoration sites, and ensure that the seed is sown at the right time to support germination during favorable conditions for establishment.

Many species produce dormant seeds to control the timing of germination to ensure emergence when there is the greatest probability of survival and growth, particularly in habitats with unfavorable, extreme, or unpredictable climates [4–6]. Dormancy break, germination, and emergence can present the first major recruitment bottlenecks in the restoration process [3,7]. This is particularly true if the seed is not appropriate for the restoration site, or if it is sown at the wrong time, which can result in the emergence of only a fraction of the sown seed. Ensuring that the seeds used in a restoration have the greatest likelihood of germination and establishment requires understanding the factors that control dormancy and germination among and within species.

Species with a similar phylogenetic history tend to share common traits [8]. Seed dormancy and germination are two traits that have been shown to be linked to phylogeny [9–11], with closely-related species often exhibiting similar germination patterns independent of geographic distribution and climatic conditions [12]. For example, species in the Fabaceae distributed worldwide typically have physical dormancy and need scarification to allow water to permeate the seed coat and break dormancy [5]. Thus, if specific dormancy-breaking requirements are not known for a species, it can be useful to look to other species in the same genus or family for guidance on how to alleviate dormancy. However, there are many cases in which closely-related species do not have the same germination response [13] or even dormancy classification (e.g., not all Fabaceae have physically dormant seeds [5,14]).

In some cases, a lack of congruency in dormancy types among related species may be driven by adaptation to environmental conditions. Species have adapted to their environment through different types of seed dormancy regulation since the origin of seed plants [15]. Within species, populations may adapt to their home environment [16,17], with local populations outperforming non-local populations when grown under local environmental conditions [18–20]. Dormancy is a highly heritable trait and under strong selective pressure, because it directly determines the conditions to which a seedling will be exposed [21,22]. Once dormancy is broken, and germination has started, the embryo is irreversibly committed to growth. If dormancy breaks in the wrong conditions, it can result in lack of germination and/or seedling mortality [7]. Temperature and precipitation are two major selective forces involved in breaking dormancy and inducing germination [23], and they are perhaps the most important environmental variables that cue germination and thus affect subsequent seedling establishment in suitable climatic conditions [22,24]. Research has shown that within-species variation in seed dormancy and germination is often correlated with environmental gradients imposed by latitude [25], elevation [26,27], and temperature [25,28,29], and can also be explained by habitat type [30,31].

Only a few studies have begun to disentangle how phylogenetic history and local environmental conditions interact to influence seed dormancy and germination [32,33]. They have found that phylogeny, environmental conditions, and habitat all contribute to variance in germination, with phylogeny explaining the highest percentage of variance. However, these studies focus on differences at the species level, ignoring potential variation at the population level that may be driven by adaptation to divergent environmental conditions. This information is needed to understand whether seed sourcing and seasonal seeding timing recommendations for restorations can be made at the species level, or to what degree they need to be tailored to specific seed sources and restoration sites [7,34,35].

We build on previous phylogenetic and environmental studies of seed dormancy and germination to investigate seed germination patterns among multiple populations of eight forb species native to the highly heterogeneous landscape of the southwestern United States. Germination responses were evaluated under multiple treatments designed to mimic climatic conditions throughout the region. This allows us to examine how the phylogenetic history of each species and the climatic conditions of each seed source site explain variation in seed dormancy and germination patterns. We expect that variation in germination requirements within and between species will be significantly explained by source climate as well as phylogenetic relatedness. We discuss how our results can be applied to restoration-relevant questions of seed sourcing and seed application timing.

## Methods

### Ethics statement

Permits to collect seed for this study came from the Bureau of Land Management (BLM, contact person UT: Rachel Hosna; contact person AZ: Troy Wood; contact person NM: Zoe Miller). Multiple collections (including all collections from CO) were sent through colleagues associated with the BLM Seeds of Success program and the Uncompahgre Partnership. Seed was not collected from any endangered or protected species.

### Study system

**Fig 1:**
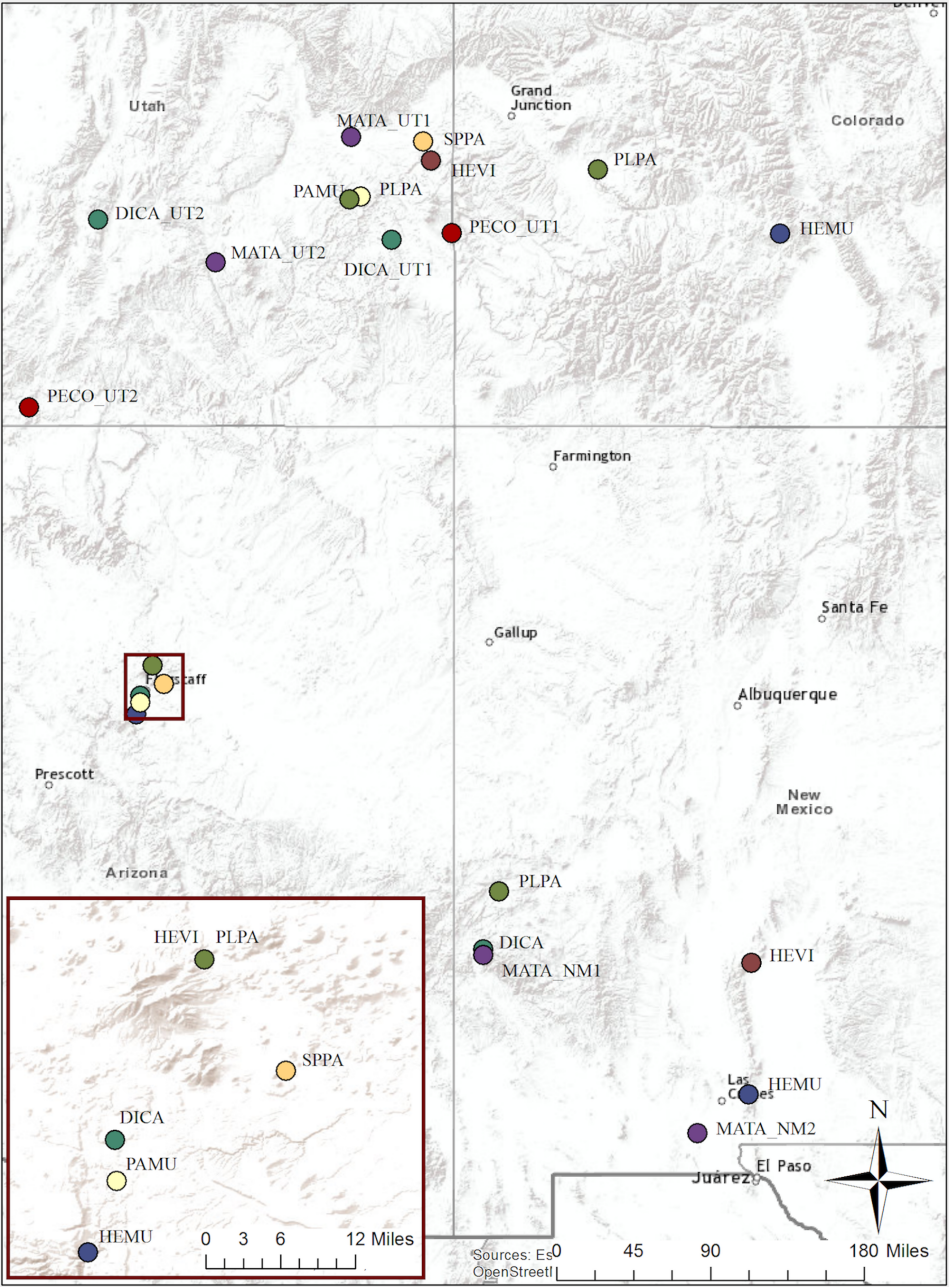
Seed collection locations. Species names are abbreviated with a species code and population number if there are multiple populations within one state. Each species has a unique color. The insert shows the Flagstaff area at a larger scale. Population abbreviations correspond to labels in Table 1.

The species used in this study are native to the semi-arid habitats of the southwestern United States, including the states of Colorado, Utah, Arizona, and New Mexico (Fig 1). The region is topographically diverse, and climatic conditions of most of the region are classified as semi-arid and fluctuate from hot summers with monsoonal rains in the south to cold winters with substantial snowfall in the north and at higher elevations [36]. Total annual precipitation is approximately 250 mm/year, with drier areas receiving as little as 130 mm/year, and high elevations receiving as much as 670 mm/year [37]. Winter and summer precipitation can be highly variable from year to year [38]. Ecosystems of the region include red rock deserts, high elevation plateaus, woodlands, sagebrush shrublands, salt desert shrublands, and mountain peaks [36]. This is a region of increasing restoration priority, yet regionally-sourced plant material is rarely available for restorations [36,39].

### Species and population sampling

The eight native forb species selected for this study are common and widespread throughout the study region, and are considered high priority species for use in large-scale restorations in the region [39]. We selected the species with the aim of including multiple families and multiple genera within families to capture a range of phylogenetic relatedness. The focal species are: *Dieteria canescens* (Pursh) Nutt. (Asteraceae; formerly *Machaeranthera canescens* [40]), *Heliomeris multiflora* Nutt. (Asteraceae), *Heterotheca villosa* (Pursh) Shinners (Asteraceae), *Machaeranthera tanacetifolia* (Kunth) Nees (Asteraceae), *Packera multilobata* (Torr. & A. Gray ex A. Gray) W.A. Weber & Á. Löve (Asteraceae), *Penstemon comarrhenus* A. Gray (Plantaginaceae), *Plantago patagonica* Jacq. (Plantaginaceae), and *Sphaeralcea parvifolia* A. Nelson (Malvaceae). Only two study species have been classified by dormancy type: *H*. *villosa* (non-dormant) and *P*. *patagonica* (physiologically dormant; requires a period of warm and/or cold stratification to break dormancy) [41]. It is likely that the other species in Asteraceae and Plantaginaceae have physiological dormancy (PD) or are non-dormant, while species in Malvaceae can have physiological and/or physical dormancy (PY; requires mechanical or chemical scarification before breaking dormancy) [41]. Previous research on the Malvaceae species used in this study, *S*. *parvifolia*, suggests that this species has combinational dormancy (PD + PY) [42].

Populations of all species were sampled from at least two source sites in the study area to achieve a sampling of the different climatic conditions found throughout the Southwest region (Fig 1 and Table 1). Populations were located using known location information, Southwest Environmental Information Network herbarium records from Arizona State University Vascular Plant Herbarium, Deaver Herbarium (Northern Arizona University), and San Juan College Herbarium [43], and other records/personal communication. Seeds were collected in the summer and fall of 2015 from two to four populations for each species, for a total of 24 populations across the states of Colorado, Utah, Arizona and New Mexico. Seeds of Success collection protocols were followed, targeting large populations and creating a bulked collection of ripe seed collected from at least 30 individuals [44]. Seeds were cleaned to remove debris at the Chicago Botanic Garden, and stored at room temperature for two weeks following collection to allow for after ripening. After two weeks, seeds were placed in dryers at 15°C and 15% relative humidity until germination trials began, which varied based on the start of stratification treatments between January and March 2016.

**Table 1:** Collection information for the study species.

Species (code)	Family	Lifespan	State	Latitude	Longitude	MAT	AP
*Dieteria canescens*	Asteraceae	A/B/P					
(DICA)							
			UT (2)	38.38	-112.05	4.1	381
			AZ	35.16	-111.70	7.6	564
			UT (1)	38.25	-109.57	9.3	322
			NM	33.39	-108.80	9.3	483
*Heliomeris multiflora*	Asteraceae	P					
(HEMU)							
			CO	38.29	-106.29	1.9	422
			AZ	35.03	-111.73	10.4	556
			NM	32.36	-106.55	12.4	363
*Heterotheca villosa*	Asteraceae	P					
(HEVI)							
			AZ	35.37	-111.59	6.2	540
			NM	33.29	-106.53	8.8	506
			UT	38.77	-109.24	11.3	267
*Machaeranthera tanacetifolia*	Asteraceae	A/B					
(MATA)							
			NM (1)	33.34	-108.80	7.8	529
			UT (1)	38.93	-109.92	11.2	210
			UT (2)	38.10	-111.06	11.6	177
			NM (2)	32.08	-106.99	16.0	240
*Packera multilobata*	Asteraceae	A/P					
(PAMU)							
			AZ	35.11	-111.70	8.2	564
			UT	38.53	-109.83	10.1	266
*Penstemon comarrhenus*	Plantaginaceae	P					
(PECO)							
			UT (1)	38.29	-109.06	6.8	427
			UT (2)	37.13	-112.64	9.3	390
*Plantago patagonica*	Plantaginaceae	A					
(PLPA)							
			AZ	35.37	-111.59	6.2	540
			NM	33.79	-108.67	9.1	387
			CO	38.71	-107.83	9.3	294
			UT	38.52	-109.93	11.1	228
*Sphaeralcea parvifolia*	Malvaceae	P					
(SPPA)							
			UT	35.24	-111.50	8.9	465
			AZ	38.90	-109.31	12.0	229

Numbers in parentheses correspond to species ID codes found in figures. Lifespan codes: A = annual, B = biennial, P = perennial. MAT = mean annual temperature (°C); AP = annual precipitation (mm). Populations within species are arranged from coolest to warmest based on MAT.

### Germination study

Seeds were x-rayed [45] prior to the start of germination trials to estimate the proportion of filled seeds in each collection, which has been used as an effective maximum estimate of viability [46]. Seeds that were x-rayed were not used in the experiment. X-raying allowed us to discern between seeds that likely had a full embryo (and were presumably viable) and those that did not from visual inspection alone. Immediately before plating, the seeds were sterilized in 5% sodium hypochlorite (bleach) for 30 seconds and rinsed two times in sterile deionized water. Seeds were then placed on 1.5% agar in 15 × 60 mm petri plates, and exposed to stratification treatments as follows: 1) no stratification; 2) 8 weeks at 3°C (in dark refrigeration; simulating a long winter and spring germination); 3) 3 weeks at 3°C (in dark refrigeration; simulating a short winter and spring germination); and 4) 3 weeks at 30°C (in dark, climate-controlled incubation [47]; simulating a short summer monsoon and fall germination) [29]. *Sphaeralcea parvifolia* has been shown to have combinational dormancy (physiological + physical dormancy), and was therefore scarified (i.e., mechanical scarification by sandpaper; lightly rubbed between sandpaper for 15–30 seconds, or until the seed coat appeared broken) after bleaching and prior to stratification. Following stratification, half the seeds from each treatment were incubated under 20/10°C (simulating spring or fall germination temperatures) and half were incubated under 25/15°C (simulating summer germination temperatures) with an alternating 12 hr. dark/12 hr. light regime for 18 days using climate-controlled incubators [41,47], for a total of eight separate treatments. Each population had four replicates of 25 seeds/petri plate for each stratification by incubation treatment. Petri dishes were randomized inside the incubators, and examined every 48 hours for germinated seeds throughout the experiment. Seeds with a radicle extending 1mm were considered germinated and removed from the petri plate. Seeds that did not germinate after 18 days in incubation were removed and subjected to a ‘cut test’ to determine fill and thereby likely viability, which was used in viability adjusted germination analyses [41]. Seeds that were filled with a white intact embryo were considered viable.

### Statistical analyses

The total proportion of germinated seeds at the end of the study was used to construct a data frame of germination traits per population, in which each population had eight traits (mean germination proportion under each of the eight treatments, including all seeds that germinated in cold stratification as well as incubation). Additionally, because there was high germination during stratification for many populations, a data frame of only stratification traits per population was created, in which each population had three traits (mean germination proportion in each of the three stratification treatments). All analyses were performed on viability-adjusted germination and executed using R: A Language and Environment for Statistical Computing [48].

To determine the influence of phylogenetic relatedness and climate on germination responses, Mantel tests were conducted with the ecodist package [49], using distance matrices of germination traits, phylogenetic branch distances, and climatic data. To produce a distance matrix for phylogenetic relationships among species, a phylogenetic tree was created using Phylomatic version 3 using the stored tree from Zanne et al. 2014 [50,51]. The branch lengths of the tree, in millions of years, were used to create a distance matrix with cophenetic distances.

Distance matrices were generated for the germination trait data (with separate matrices for total germination and germination during stratification) and climatic data using Euclidean distances. Climatic data for seven bioclimatic variables were extracted for each population using raster information from WorldClim and the ‘biovars’ function in the dismo package [52]. The bioclimatic variables included BIO1: Mean Annual Temperature, BIO2: Mean Diurnal Range (max temp-min temp), BIO4: Temperature Seasonality 7, BIO8: Mean Temperature Wettest Quarter, BIO12: Mean Annual Precipitation, BIO15: Precipitation 8 Seasonality, and BIO18: Precipitation Warmest Quarter. These seven variables represent orthogonal axes of climatic variation across the Southwest [53,54]. The bioclimatic data were log transformed prior to creating a distance matrix to ensure values were evenly scaled.

The trait distance matrices (total germination and germination during stratification) were tested separately for significant correlations with the phylogenetic and the climatic distance matrices, respectively, using Mantel tests with 10000 permutations. Mantel tests were also performed with the two separate trait distance matrices and a distance matrix based on the four climatic variables that correspond to temperature (BIO1, BIO2, BIO4, and BIO8), as well as a distance matrix based on the three climatic variables that correspond to precipitation (BIO12, BIO15, and BIO18). Finally, a distance matrix of each of the seven individual bioclimatic variables was tested against the two trait distance matrices to determine if any one variable significantly explained germination differences.

To examine whether the final germination response within species differed across treatments and populations, we used two approaches. First, we fit a Bayesian generalized linear mixed model (GLMM) that accounted for phylogenetic relatedness among species. We then fit a general linear model (GLM) for each species individually using AIC model selection.

To account for phylogenetic relatedness among species, we fit a Bayesian generalized linear mixed model (GLMM), with observations assumed to be Binomially-distributed with the logit link function. The germination probability for the *j*th population of the *i*th species under the experiment with stratification length, *sl*, stratification temperature, *st*, and incubation temperature, *it*, was given by

$$\begin{array}{l} \mathrm{logit}\left(p_{i,j,st,sl,it}\right)=\beta_{i}^{S}+\delta_{i}^{P}\beta_{ij}^{P}\\ \;+\delta_{i}^{\mathrm{SL}}\beta_{i,sl}^{\mathrm{SL}}+\delta_{i}^{\mathrm{ST}}\beta_{i,st}^{\mathrm{ST}}+\delta_{i}^{\mathrm{IT}}\beta_{i,it}^{\mathrm{IT}}\\ \;+\delta_{i}^{P:\mathrm{SL}}\beta_{i,j,sl}^{P:\mathrm{SL}}+\delta_{i}^{P:\mathrm{ST}}\beta_{i,j,st}^{P:\mathrm{ST}}+\delta_{i}^{P:\mathrm{IT}}\beta_{i,j,it}^{P:\mathrm{IT}}\\ \;+\delta_{i}^{\mathrm{SL}:\mathrm{ST}}\beta_{i,sl,st}^{\mathrm{SL}:\mathrm{ST}}+\delta_{i}^{\mathrm{SL}:\mathrm{IT}}\beta_{i,sl,it}^{\mathrm{SL}:\mathrm{IT}}+\delta_{i}^{\mathrm{ST}:\mathrm{IT}}\beta_{i,st,it}^{\mathrm{ST}:\mathrm{IT}}. \end{array}$$

The indicators, *δ*, were used to include or exclude the population and treatment factors and their interactions from the model. Each was given a prior probability of *P*(*δ* = 1) = 0.5, with interaction indicators conditioned on the inclusion of their interacting factors in the model. Population-dependent effect sizes, *β*, were all given N(0,1) priors. We accounted for phylogenetic correlations by modeling the remaining (non-population-dependent) coefficients, *β*, as multivariate normally–distributed with the mean sampled from a N(0,1) (or fixed at 0 for interaction effects) and covariance given by *σ*^2^**A**, where **A** is the phylogenetic relatedness matrix [55] calculated with MCMCglmm [56]. While **A** was shared, a separate *σ*^2^ was fitted for each coefficient with a gamma prior distribution 1/*σ*^2^ ∼ *Γ*(0.1,0.1).

We implemented the model in JAGS v4.3.0 with the GLM module [57] to sample all parameters from the posterior, using 4 chains each with 5000 iterations of adaptation and another 5000 iterations of burnin before taking 50,000 samples, for a total of 200,000 samples. The analysis was run twice, first considering all of the germination data and then using only germination during stratification.

To examine intraspecific germination response without accounting for phylogenetic relatedness, generalized linear models with binomial error and logit link function were fitted to germination results for each species using the three treatment factors (stratification length, stratification temperature, and incubation temperature) and population as predictors. The ‘step’ function was used to find all possible model iterations for each species using AIC model selection with backward and forward elimination. The same ‘maximal’ model was used for each species to begin the AIC model selection process. All possible model iterations across all species were used as candidate models to generate an AIC table and determine the best-fit model for each species. Models with a delta AIC of less than two were considered good candidate models, but the model with the lowest AIC value and greatest AIC weight was used for subsequent analysis and creating figures.

The above methods were also used to generate generalized linear models for germination in stratification. Germination was used as the response with population, stratification length, and stratification temperature as predictors.

## Results

The Mantel tests showed that phylogenetic relatedness and source climatic conditions are both significantly correlated with overall germination response. Germination traits were significantly correlated with phylogenetic distance (r = 0.3; p = 0.008), as well as climatic distance of all bioclimatic variables (r = 0.19; p = 0.03). Additionally, germination response was correlated with climatic distance of temperature-dependent bioclimatic variables (r = 0.24; p = 0.03). Germination response was not significantly correlated with precipitation-dependent climatic variables or any one individual bioclimatic variable.

In all eight species, some seeds germinated during cold or warm stratification, *i*.*e*., in darkness at 3°C or 30°C, respectively. The Mantel tests performed on germination during stratification showed that germination traits were not significantly correlated with overall climate (r = 0.005; p = 0.45), temperature-dependent variables (r = 0.009; p = 0.42), precipitation-dependent variables (r = 0.05; p = 0.19), or phylogenetic relatedness (r = -0.04; p = 0.61). However, germination traits were correlated with one bioclimatic variable, precipitation seasonality (r = 0.23; p = 0.01).

The GLMM analyses, for both overall germination and germination during stratification, found that there was 0 posterior probability that the results were entirely explained by phylogeny. A model that used at least one population predictor was always preferred. Seven of the eight species showed significant variation among populations and significant interaction of population with at least one treatment factor in overall germination (Table 2). Seven of the eight species showed significant variation among populations when looking at germination during stratification, and six of the eight species showed a significant interaction between population and at least one treatment factor (Table 3).

**Table 2:** Results from MCMCglmm analysis on overall germination.

Species	P	SL	ST	IT	P:SL	P:ST	P:IT	SL:ST	SL:IT	ST:IT
*Dieteria canescens*	1.00	1.00	1.00	1.00	0.09	0.69	1.00	0.46	0.80	1.00
*Heliomeris multiflora*	1.00	1.00	1.00	1.00	1.00	1.00	1.00	0.42	0.14	0.07
*Heterotheca villosa*	0.07	0.01	0.56	0.77	0.00	0.00	0.00	0.00	0.00	0.53
*Machaeranthera tanacetifolia*	1.00	1.00	0.95	1.00	1.00	0.94	1.00	0.44	0.34	0.75
*Packera multilobata*	0.99	0.30	0.92	0.76	0.14	0.52	0.25	0.11	0.10	0.62
*Penstemon comarrhenus*	1.00	1.00	1.00	1.00	1.00	0.21	0.08	0.44	0.47	1.00
*Plantago patagonica*	1.00	1.00	1.00	1.00	0.70	1.00	1.00	0.48	0.67	0.71
*Sphaeralcea parvifolia*	1.00	1.00	0.94	0.95	1.00	0.34	0.13	0.37	0.22	0.11

The table shows the posterior probability that each predictor belongs in the model for each species. P = population, SL = stratification length, ST = stratification temperature, IT = incubation temperature.

**Table 3:** Results from MCMCglmm analysis on germination during stratification.

Species	P	SL	ST	IT	P:SL	P:ST	P:IT	SL:ST	SL:IT	ST:IT
*Dieteria canescens*	1.00	1.00	1.00	1.00	0.10	0.92	1.00	0.46	0.59	1.00
*Heliomeris multiflora*	1.00	1.00	1.00	1.00	1.00	1.00	1.00	0.45	0.29	0.17
*Heterotheca villosa*	0.05	0.02	0.74	0.96	0.00	0.01	0.01	0.01	0.01	0.69
*Machaeranthera tanacetifolia*	1.00	0.98	1.00	1.00	0.76	1.00	1.00	0.50	0.19	1.00
*Packera multilobata*	1.00	0.25	0.95	0.79	0.10	0.41	0.33	0.10	0.09	0.68
*Penstemon comarrhenus*	1.00	1.00	0.94	0.96	1.00	0.28	0.10	0.39	0.29	0.93
*Plantago patagonica*	1.00	1.00	1.00	1.00	1.00	1.00	1.00	0.49	0.52	0.35
*Sphaeralcea parvifolia*	1.00	1.00	0.96	0.59	1.00	0.52	0.07	0.40	0.17	0.09

The table shows the posterior probability that each predictor belongs in the model for each species. P = population, SL = stratification length, ST = stratification temperature, IT = incubation temperature.

**Fig 2:**
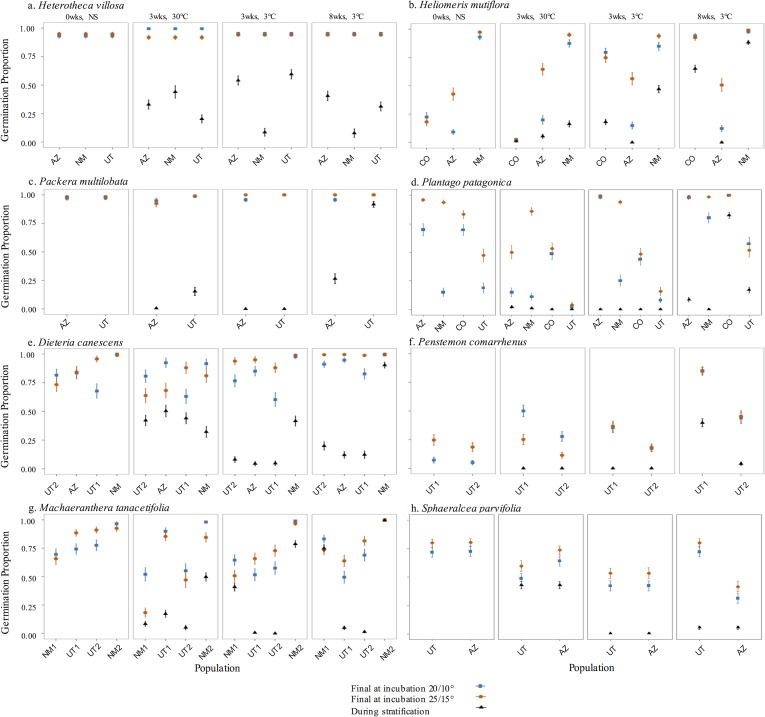
Final germination proportion and germination during stratification for each species. Results are based on best-fit GLMs with treatment factors and population as predictors (binomial or quasibinomial error with logit link function). Standard error bars are displayed. Populations within species are arranged from cooler populations on the left to warmer populations on the right, based on mean annual temperature. (a) *Heterotheca villosa*; (b) *Heliomeris multiflora*; (c) *Packera multilobata*; (d) *Plantago patagonica*; (e) *Dieteria canescens*; (f) *Penstemon comarrhenus*; (g) *Machaeranthera tanacetifolia*; (h) *Sphaeralcea parvifolia*.

The results of the generalized linear models for each species were very similar to those of the GLMMs, whereby the final germination response significantly varied by population for seven of eight species (Fig 2 and Table 4). *Heterotheca villosa*, which had an average germination proportion of 0.95 ± 0.005 across all treatments and populations, was the only species in which population was not a significant predictor of germination response. Population variation was significant, but relatively small for some species. For example, the best-fit model for *Packera multilobata* included population as a significant predictor, but germination proportion differences between populations were very small, with an average germination proportion of 0.98 ± 0.004 across all treatments and populations. Other species, including *Heliomeris multiflora* and *Plantago patagonica*, showed dramatic differences between populations and within and across treatments.

**Table 4:** Results from model selection using AIC on final germination.

	*k*	AIC	ΔAIC	AICwt
***Dieteria canescens***				
P+SL+ST+IT+P:ST+P:IT+SL:IT+ST:IT	20	476.91	0	0.50
P+SL+ST+IT+P:ST+P:IT+ST:IT	19	478.47	1.57	0.23
P+SL+ST+IT+P:SL+P:ST+P:IT+SL:IT+ST:IT	23	478.81	1.90	0.19
***Heliomeris multiflora***				
P+SL+ST+IT+P:SL+P:ST+P:IT	15	349.15	0	0.71
***Heterotheca villosa***				
SL+ST+IT+SL:IT+ST:IT	8	223.21	0	0.51
P+SL+ST+IT+SL:IT+ST:IT	10	224.12	0.91	0.32
***Machaeranthera tanacetifolia***				
P+SL+ST+IT+P:SL+P:ST+P:IT+ST:IT	22	512.10	0	0.66
P+SL+ST+IT+P:SL+P:ST+P:IT+SL:IT+ST:IT	23	513.51	1.41	0.32
***Packera multilobata***				
P+ST+IT+P:ST+P:IT+ST:IT	10	109.56	0	0.42
P+SL+ST+IT+P:ST+P:IT+ST:IT	11	110.91	1.35	0.21
***Penstemon comarrhenus***				
P+SL+ST+IT+P:SL+ST:IT	10	277.61	0	0.26
P+SL+ST+IT+P:SL+P:ST+ST:IT	11	277.86	0.25	0.23
P+SL+ST+IT+P:SL+SL:IT+ST:IT	11	278.22	0.62	0.19
P+SL+ST+IT+P:SL+P:ST+SL:IT+ST:IT	12	278.45	0.85	0.17
***Plantago patagonica***				
P+SL+ST+IT+P:SL+P:ST+P:IT+SL:IT+ST:IT	23	503.40	0	0.50
P+SL+ST+IT+P:SL+P:ST+P:IT+ST:IT	22	504.71	1.31	0.26
P+SL+ST+IT+P:SL+P:ST+P:IT+SL:IT	22	504.87	1.47	0.24
***Sphaeralcea parvifolia***				
P+SL+ST+IT+P:SL+P:ST	9	295.38	0	0.34
P+SL+ST+IT+P:SL+P:ST+P:IT	10	296.28	0.90	0.22

Evaluation of the effects of treatment factors and population on germination response for each species. Only candidate models with a delta AIC of less than 2 are shown. P = population, SL = stratification length, ST = stratification temperature, IT = incubation temperature.

Population significantly interacted with at least one treatment factor in seven of the eight species (Table 4). Populations of *H*. *villosa* did not interact with any treatment factors to predict germination response. Populations of *H*. *multiflora*, *Machaeranthera tanacetifolia*, and *P*. *patagonica* interacted significantly with all three treatment factors (stratification length, stratification temperature, and incubation temperature), and showed the greatest variation between populations and treatments.

Generalized linear models for germination response in stratification showed that population was significant for all eight species (Fig 2 and Table 5). Furthermore, population significantly interacted with at least one treatment factor (stratification length or stratification temperature) in all species.

**Table 5:** Results from model selection using AIC on germination in stratification.

	*k*	AIC	ΔAIC	AICwt
***Dieteria canescens***				
P+SL+ST+P:SL+P:ST	12	419.22	0	1
***Heliomeris multiflora***				
P+SL+ST+P:ST	7	213.76	0	0.86
***Heterotheca villosa***				
P+SL+ST+P:SL+P:ST	9	301.26	0	0.67
P+SL+ST+P:ST	7	302.63	1.37	0.33
***Machaeranthera tanacetifolia***				
P+SL+ST+P:SL+P:ST	12	278.35	0	0.95
***Packera multilobata***				
P+SL+ST+P:ST	5	145.98	0	0.42
P+SL+ST+P:SL	5	145.98	0	0.42
***Penstemon comarrhenus***				
P+SL	3	65.02	0	0.59
***Plantago patagonica***				
P+SL+ST+P:ST	9	147.51	0	0.48
P+SL+ST+P:SL	9	147.51	0	0.48
***Sphaeralcea parvifolia***				
P+SL+ST+P:SL	5	125.06	0	0.54
P+SL+ST+P:SL+P:ST	6	125.98	0.92	0.34

Evaluation of the effects of treatment factors and population on germination response for each species during stratification. Only candidate models with a delta AIC of less than 2 are shown. P = population, SL = stratification length, ST = stratification temperature.

## Discussion

Our findings show that phylogenetic distance between species significantly explains thirty percent of the variation in final seed germination, which indicates constraints on seed dormancy regulation among species imposed by phylogenetic history [5,58,59]. However, a significant portion of the variation in germination response was also explained by source climate and temperature (19% of final germination variation explained by climate, 25% of final germination variation explained by four bioclimatic variables summarizing temperature, and 23% of germination during stratification explained by precipitation seasonality). These results suggest that within-species variation in seed dormancy and germination traits (including germination in stratification as well as final germination proportion) requires much more attention in seed dormancy research and restoration application.

Overall, phylogenetic relatedness significantly explains some, but not all, of the differences in germination response across populations and treatments, with closely related species exhibiting similar germination responses. These results agree with previous studies that found that phylogenetic relatedness is a significant factor contributing to variation in germination among species. For example, the germination strategies of 109 species from degraded sandy grasslands in China were significantly correlated with relatedness, dispersal strategy, and seed shape [60]. A study on 633 species native to alpine meadows in the Qinghai-Tibet Plateau found that taxonomic membership explained the majority of germination variation [61]. And finally, a study on 69 species from arid and semiarid zones in northwest China found that phylogeny and life history were the two factors that explained the majority of variance in final germination [32]. In general, germination appears to be, at least in part, constrained by evolutionary history. However, none of these studies examined population-level germination variation. Our results show that when multiple populations from diverse habitats and climates are included in species-wide investigations of dormancy and germination, both phylogeny and climate explain germination variation among and within species.

Adaptation to climatic conditions at source sites may explain why climate significantly explained variation in germination traits of our study species. Local adaptation to climatic conditions have been found in a wide range of species and habitats [18,19]. Species widely distributed throughout the varied habitats of the western United States may be particularly likely to be adapted to local climate conditions. For example, germination timing in 135 populations of 38 *Penstemon* species distributed throughout the Intermountain West was correlated with collection site climate and winter length, suggesting that multiple lineages in the genus have evolved habitat-specific germination strategies to ensure survival in harsh environments [27]. However, to explicitly test assumptions of local adaptation in our species requires reciprocal transplant studies that ideally control for potential maternal effects and directly test whether variation leads to a home-site advantage [18,62].

When investigating which aspects of climate may drive dormancy and germination patterns in our study species, we found that temperature was particularly important in explaining our final germination response (about 25% of the variation). This supports previous studies and reviews that highlight the important role that temperature plays in dormancy regulation and germination [5,24,63]. However, we also found that precipitation seasonality significantly explained variation in germination during stratification. This germination trait is likely very important for seedling survival, as it is regulated by dormancy and could either promote emergence in suitable conditions or lead to emergence in unsuitable conditions if the timing is not right. Yet this trait is rarely measured, and in the cases where it is, it is rarely analyzed as a unique response, but rather subsumed in the final germination analyses (e.g.,[64]). The potential for germination in stratification merits more explicit consideration in species and populations in which it may occur.

The yearly and multi-decadal variation in climatic conditions found throughout the southwestern U.S. has likely selected for complex dormancy and germination patterns in our study species [65]. For example, the timing of germination-triggering events, such as precipitation seasonality, is the most significant driver of germination response in the Southwest. The timing of these events was found to be a stronger driver of germination than temperature alone during the growing season [65]. Our results confirm that there is a significant relationship between final germination, climate, and temperature, but more specifically that there is a significant correlation between germination during stratification and precipitation seasonality. Northern populations, where the majority of annual precipitation falls as snow during winter months, were on average less likely to germinate in cold stratification than populations from New Mexico, which experience a seasonal monsoon during the summer [38,66,67]. This may be the result of selection acting on dormancy break, as seeds that germinate during the middle of cold, snowy winter months in northern populations are less likely to survive, while those that germinate during summer monsoons in the south may be able to take advantage of extra moisture and warm growing conditions. However, these differences would not be obvious from final germination results alone. It is therefore essential to examine dormancy and germination patterns during stratification to fully understand germination response and the relationship to climate and other factors. Thus, germination during stratification should be included as a trait in seed transfer zone research, particularly in thinking about movement between seed zones and timing of seed sowing for restoration activities.

This study is not comprehensive of all factors that may be contributing to variation in germination among and within populations, and future studies should aim to incorporate methods that would disentangle the effects of other factors. For example, the variation identified in this study could be influenced by genetic factors (local adaptation or random genetic drift), by environmental factors (maternal effects or the environment that the seeds were exposed to prior to harvest), or by storage factors (differences in dry storage length). The many factors that influence dormancy and germination patterns are quite complex. Understanding which factors are the primary drivers of patterns at the population level can influence seed sourcing for restorations. Climatic conditions at the source site have been shown to be one of the primary factors in predicting variation in germination patterns. As such, seed zone delineations should continue to use temperature and climatic data to guide movement of plant materials for restorations, particularly in the Southwest, where the climate and terrain are extremely heterogeneous and fine-scale delineations are necessary [68].

### Restoration implications

Seed germination can lead to major recruitment bottlenecks in restorations if the seed is not appropriately adapted to the restoration site [7]. Our results show that among- and within-species variation in germination can be partly explained by differences in climatic conditions between sites, which is consistent with other studies that have identified a close relationship between dormancy and germination and environmental conditions, such as temperature and precipitation. Across all species tested, temperature and precipitation seasonality were found to be the most significant climatic drivers of germination response, providing evidence for the inclusion of these variables in seed sourcing decisions in this region.

Several seed sourcing considerations emerge from our study. The first is to consider the stratification requirements of each population and the timing of seed sowing for the restoration. Populations from colder climates typically need a period of cold stratification in order to break dormancy and germinate [67,69,70], whereas many of the southern, warmer populations in this study showed little differences between cold and warm stratification treatments. Other species need a long period of cold stratification regardless of source climate, such as *Penstemon comarrhenus*.

Many populations in our study germinated during stratification and across incubation temperatures, suggesting that germination, at least under laboratory conditions, may largely respond to moisture availability. If these results translate to germination responses under field conditions, demographic bottlenecks may occur in the restoration process if seeds germinate when moisture conditions are favorable for germination, but conditions for seedling survival are not favorable. For example, a population from New Mexico that may be adapted to the monsoonal gradient and times germination to coincide with high levels of precipitation in the summer could germinate at a restoration site with high precipitation during winter months, in which case the seedling may not survive until the warm growing season. Given the negative economic and ecological consequences of using seeds that are not able to germinate or successfully establish at a restoration site, it is important to consider all aspects of seed germination in restoration seed sourcing research and decision-making.

Phylogenetic relatedness significantly explained differences in germination response. Most of the Asteraceae species in this study had high germination across all populations and treatments, which may indicate a lower likelihood of bottlenecks at the germination stage when moving seed from one site to another, climatically dissimilar site. However, these results do not necessarily translate to later life stages, such as establishment and survival, and maladaptation may become apparent later. Therefore, movement of seeds between dissimilar sites should be guided by research on how this affects all life stages. It is also worth noting that *Heliomeris multiflora* is in the Asteraceae, and it showed dramatic variation between populations and a close relationship to source site climate. It is noteworthy that this is the only Asteraceae species in our study that lacks a pappus, and is therefore gravity-dispersed, whereas all other Asteraceae species in our study have a pappus and are wind-dispersed. These results indicate that not all members of a taxonomic group will necessarily behave similarly, and this species should be sourced from climatically similar sources to ensure the greatest chances of germination and emergence.

## Conclusions

This study demonstrated the significant impact of both species relatedness and climate on germination response at the species and population level. While numerous factors may be contributing to the germination variation we identified, it is quite clear that variation in dormancy and germination is driven both by species identity and population differences. It is therefore very important to understand species-specific traits, as well as site conditions where the seed was collected and where it will be used in order to, as The National Seed Strategy states, use ‘the right seed in the right place at the right time’ [34].

## Supporting information

S1 Dataset Germination and climate data. (XLSX)
